# Quality of antenatal care and associated factors in a rural county in Kenya: an assessment of service provision and experience dimensions

**DOI:** 10.1186/s12913-019-4476-4

**Published:** 2019-10-07

**Authors:** Patience A. Afulani, Laura Buback, Francisca Essandoh, Joyceline Kinyua, Leah Kirumbi, Craig R. Cohen

**Affiliations:** 1 School of Medicine, University of California, San Francisco, USA; 2 Institute for Global Health Sciences, UCSF, San Francisco, USA; 3 University of New England, Portland, USA; 4 Kenya Medical Research Institute, Nairobi, Kenya

**Keywords:** Antenatal care, Quality of care, Person-centered care, Experience of care, Services provision, Kenya

## Abstract

**Background:**

This study aimed to assess the quality of antenatal care (ANC) women received in Migori county, Kenya—including both service provision and experience dimensions—and to examine factors associated with each dimension.

**Methods:**

We used survey data collected in 2016 in Migori county from 1031 women aged 15–49 who attended ANC at least once in their most recent pregnancy. ANC quality service provision was measured by nine questions on receipt of recommended ANC services, and experience of care by 18 questions on information, communication, dignity, and facility environment. We summed the responses to the individual items to generate ANC service provision and experience of care scores. We used both linear and logistic regression to examine predictors.

**Results:**

The average service provision score was 10.9 (SD = 2.4) out of a total of 16. Most women received some recommended services once, but not at the frequency recommended by the Kenyan Ministry of Health. About 90% had their blood pressure measured, and 78% had a urine test, but only 58 and 14% reported blood pressure monitoring and urine test, respectively, at every visit. Only 16% received an ultrasound at any time during ANC. The average experience score is 27.3 (SD = 8.2) out of a total score of 42, with key gaps demonstrated in communication. About half of women were not educated on pregnancy complications. Also, about one-third did not often understand the purposes of tests and medicines received and did not feel able to ask questions to the health care provider. In multivariate analysis, women who were literate, employed, and who received all their ANC in a health center had higher experiences scores than women who were illiterate (coefficient = 1.52, CI:0.26,2.79), unemployed (coefficient = 2.73, CI:1.46,4.00), and received some ANC from a hospital (coefficient = 1.99, CI: 0.84, 3.14) respectively. The wealthiest women had two times higher odds of receiving an ultrasound than the poorest women (OR = 2.00, CI:1.20,3.33).

**Conclusion:**

Quality of ANC is suboptimal in both service provision and experience domains, with disparities by demographic and socioeconomic factors and facility type. More efforts are needed to improve quality of ANC and to eliminate the disparities.

**Electronic supplementary material:**

The online version of this article (10.1186/s12913-019-4476-4) contains supplementary material, which is available to authorized users.

## Background

Maternal and neonatal mortality has remained high in low resource settings despite progress in recent years. In 2015, about 303,000 women died from pregnancy-related causes and 2.7 million babies died during the first 28 days of life globally [1, 2]. About 2.6 million babies were also stillborn [3]. High quality antenatal care (ANC) can reduce maternal and neonatal morbidity and mortality and stillbirths through prevention, as well as early identification and management of pregnancy complications or pre-existing conditions [4]. High quality ANC can also influence women’s health seeking behavior towards choosing skilled care at birth and helping them prepare to be able to access it [5–7]. A positive experience during both pregnancy and childbirth are key to person-centered care and the right of every childbearing woman, as highlighted in recent World Health Organization (WHO) recommendations [8–10]. While the specific recommendations for frequency of ANC has varied, WHO has consistently recommended that all pregnant women receive some ANC during their pregnancy [4, 8, 11].

Kenya’s National Guidelines for Quality Obstetrics and Perinatal Care that were in use at the time of this work were based on the WHO recommendations on Focused Antenatal Care [12], which recommended four comprehensive and targeted visits. The guidelines, however, urged providers to view each visit as if it were the only visit a woman may make. The recommended content of each visit includes blood pressure and fetal growth monitoring, urine testing, Iron and Folic Acid supplementation, Tetanus Toxoid immunizations, at least two doses of Intermittent Preventative Treatment for malaria in pregnancy in endemic malaria areas and deworming after the first trimester. The first ANC visit also includes a more in-depth medical history and physical examination, including head to toe examination, recording weight and height, blood group typing, HIV testing and counseling, and assessing needs for specialized care [13]. The Kenya guidelines do not mention ultrasound scanning for routine ANC, but it is recommended for fetal assessment, including for dating, among women with preterm labor and those with complications. Early ultrasound increases the accuracy of gestational age assessments and current WHO guidelines recommend one ultrasound scan before 24 weeks of gestation [8].

The Kenya guidelines also recommend comprehensive health promotion education, with a question and answer session also recommended during each visit. These guidelines also emphasize the importance of patient experience components such as communication, respect, and dignity. It states that *“Antenatal care should be simpler, safer, friendly and more accessible. Women are more likely to seek and return for services if they feel* ***cared for and respected*** *by their providers. This personalized approach requires health care providers to use excellent interpersonal skills* ***since listening to client’s concerns is just as important as giving advice.*** *It respects clients’ right to* ***dignity, privacy, confidentiality, full and accurate information****”* [13]*.* Likewise, the most recent WHO recommendations *on antenatal care for a positive pregnancy experience*, updated in 2016*,* prioritizes person-centered care and overall well-being of the mother and baby [8].

Until recently, most prior research on maternal health care focused on use of services with research on ANC mostly on timing and frequency of ANC visits [14–17]. Increasing recognition of the role of poor-quality care to the poor maternal and neonatal outcomes has stimulated interest in assessing quality of maternal health service. However, most of the attention has focused on quality of care during childbirth. [18, 19]. Little research thus exists on quality of ANC, and most of the studies on ANC quality have focused on the receipt of recommended ANC services [20–22]. This is despite a global movement advocating for measurement and interventions to improve respectful maternity care. Only a few studies in Kenya, based on the Service Provision Assessment data at the national level, have examined multiple dimensions of quality of care. These studies suggest sub-optimal ANC quality in structural, service provision, and experience measures [22–24].

More studies, including studies at sub-national levels examining multiple dimensions of ANC quality, are critical to (1) provide information on strengths and gaps in ANC quality and (2) guide interventions in specific areas to improve provision of services and person-centered antenatal care (PCANC). This paper extends the evidence in this area. We aimed to assess levels of quality of ANC, including both service provision and experience of care, in a rural county in western Kenya. Service provision here refers to receipt of recommended evidence-based services for ANC per WHO and Kenya guidelines. Experience of care captures items related to effective communication, respect, dignity, and emotional support per the WHO framework for quality of maternal and newborn health [10]. These experience dimensions assess PCANC. We also examine factors associated with each dimension of quality of care to identify sources of disparities in quality of care.

## Methods

### Setting and data collection

The data are from a cross-sectional survey conducted with women who had recently given birth as part of a study on community perceptions of quality of maternity care in Migori county. The setting and data collection methods have been previously described [25, 26]. Briefly, Migori County is a rural county in western Kenya with 8 sub-counties and a population of about one million people [27]. The county has one referral hospital, seven sub-county hospitals, 18 health centers, several dispensaries, and a few faith-based and private health facilities [28]. The estimated total fertility rate for the county is 5.2 [29]. According to the 2014 Kenya Demographic and Health Survey, 96% of women with a live birth in the preceding five years received at least one ANC from a skilled provider, but only 56% received the recommended four-plus visits [29].

The survey was conducted in August and September 2016 with women aged 15–49 years who delivered in the nine weeks preceding the survey. A multistage sampling approach was used to select women from each sub-county in health facilities and in communities, with a target of interviewing at least 100 women from each sub-county. The interviews were conducted in English, KiSwahili, and DhLuo in private spaces in health facilities or in the homes of the respondents. About one thousand women (*N* = 1051) were interviewed, with a response rate above 98%. Ethical approval for the study was provided by the institutions listed in the ethics statement, and all participants provided written informed consent after receiving information about the research. We use data from women who received ANC at least once during their most recent pregnancy (*N* = 1031). Women received ANC from government facilities (the referral hospital, health centers, and dispensaries) as well as private facilities.

### Measures

#### Dependent variables (outcomes): measures of quality of ANC

Quality of ANC is measured by several questions asked to women to assess different aspects of the content of services they received and the nature of their interactions with providers during ANC visits (Additional file 1). The measures explored capture of both service provision and experience of care.

##### Service provision

The service provision measures are nine items asking whether they received various services for screening and prevention listed in Table 2. The questions include whether they had their height, weight, and blood pressure measured, whether they had urine and blood tests, and whether they received tetanus injections, iron supplements, antihelminthes, and antimalarials. These questions were adapted from the ANC questions in the Demographic and Health Surveys. Five of the questions have binary responses (No = 0 and Yes = 1); three have responses on a four-point frequency scale (No never = 0, Yes, a few times = 1, and Yes, most of the time = 2, and Yes, all the time = 3); and one is on a three-point frequency scale ((No = 0, Yes, once = 1, and Yes, more than once = 2). These items have factor loadings of >.2 on one factor and a reasonable Cronbach alpha of 0.5. To examine the associated factors, the response codes for the nine items are summed to create a service provision index. The summative score has a range of 0 to 16 ((5*1) + (3*3) + (1*2)). ‘Don’t know’ response options are recoded to missing before summing the responses. Ultrasound screening is examined separately as a binary variable because it was not routinely recommended and did not load well with the rest of the items.

##### Experience of care

The experience of care measures are also listed in Table 3. These questions capture mostly information sharing and communication: told results after she has been weighed, her blood pressure taken, and after urine and blood tests; educated on pregnancy complications, what to do in the event of a complication, what to expect during the pregnancy, birth preparedness, diet, and breastfeeding. Furthermore, they asked whether she understood the purpose of tests and medicines, whether she was asked if she had questions, and if she felt able to ask questions. There are also three questions capturing dignity and respect as well as one question on cleanliness of the facility. These questions were adapted from the ANC questions in the Demographic and Health Surveys, as well as from the person-centered maternity care scale [26]. Six of the questions have binary responses (No = 0 and Yes = 1), and 12 have responses on a four-point frequency scale (No never = 0, Yes, a few times = 1, and Yes, most of the time = 2, and Yes, all the time = 3). The items load together on one factor with loadings greater than 0.3 and have Cronbach alpha of 0.81. The response codes to the 18 items are summed to create an experience of care index to examine associated factors. The summative score has a range of 0 to 42 ((6*1) + (12*3)). ‘Don’t know’ response options are recoded to missing before summing the responses. We also included two items (being asked for bribe and feeling they were treated differently based on certain attributes) to assess predictability and transparency of payments and perceptions of discrimination. However, these two are not included in the index because they loaded poorly with the rest of the items in the group in the factor analysis.

#### Independent variables (predictors)

Based on prior studies and theoretical rationale, we examined various factors that might affect the quality of ANC a woman receives, including: socioeconomic and demographic factors, women’s health status, familiarity and extent of contact with the health system, and facility and provider characteristics. The demographic factors included are age, marital status, parity, tribe, and religion [20–23].

The key independent variables are the socioeconomic factors which capture a woman’s personal status and empowerment and her status based on her household or partner. These included *Employment status*, type of *Occupation*, *Education, and Literacy,* which capture economic empowerment (access to and control over the means to make a living, and receiving the material benefits of this access), cognitive and psychological empowerment (includes knowledge about rights, self-esteem, and self-efficacy) [30–32]. We also included two composite measures of empowerment: *participation in household decision-making* and *attitudes towards domestic violence* to measure sociocultural empowerment (gender norms, including norms against gender-based violence) [25, 33]. We hypothesized that more empowered women will receive higher quality care. In addition, we included experience of domestic violence, which may be associated with both empowerment and mistreatment [25, 34]. Other measures of socioeconomic status (SES) included are *Household wealth* (measured in quintiles, calculated from a wealth index based on 13 questions on household assets [35] and *Partner’s education and occupation*. In addition, we included a variable on whether they or someone in their household works in a health facility, as this could influence the type of care they receive.

Variables to capture health status related factors that might affect the care women receive include whether they had complications in the index or prior pregnancy and their own assessment of the severity of the complications (whether they felt the complication was severe or not), as well as reason for seeking ANC (for a problem or checkup). For familiarity with and extent of contact with the health system, we included a variable on whether they had received ANC in prior pregnancy, whether they had previously delivered in a health facility, and the timing and frequency of ANC. For facility and provider characteristics, we included two variables on the type of facility the woman received care in and the type of provider. Because a woman could receive care from more than one type of facility and provider, these were recoded into the highest type of facility and provider (e.g., if they received care from a hospital and a health center, it is coded as hospital, and if they received care from a doctor and nurse, it is coded as a doctor). Finally, we controlled for the timing and setting of the interview, as this might affect their responses.

### Analysis

We first ran descriptive analysis to examine the characteristics of the sample and the distribution of all ANC quality related variables using means for continuous variables and proportions for categorical variables. Next, we assessed which of the ANC quality related variables could be grouped together to generate the ANC service provision and experience of care indices using exploratory factor analysis and Chronbach’s alpha for internal consistency. We then summed the selected indices to generate ANC service provision and experience of care scores, which we used as the outcome variables in bivariate and multivariate analysis.

Because the summative scores are approximately normally distributed, we used them as continuous variables and examined mean differences in scores by the various predictors, as well as unadjusted and adjusted ordinary least squares (linear) regressions. The coefficients from the linear regressions quantify the change in scores for each unit change in continuous predictors or the difference in scores between the reference category and other categories for categorical predictors [36]. We used logistic regressions to examine the factors associated with receipt of an ultrasound. Given ultrasound is recommended for women with complications, we were particularly interested in whether women with complications, as well as certain risk factors are more likely to get an ultrasound. In addition, we dichotomized the service provision and experience of care scores around the median into low and high quality and examined them in logistic regressions for sensitivity analysis. For the multivariate analysis, we first included the key independent variables, then all of the variables that were significantly associated with the outcome measures in the bivariate analysis, in the multivariate models. We then conducted post estimation tests to assess model fit and checked for collinearity and removed variables that did not improve the models or were highly correlated with other variables in the model.

## Results

### Descriptive

Table 1 shows the characteristics of the sample. The average age was 25 years, and about 17% were less than 20 years old. Approximately 79% were married, with average parity of 3; 30% had 4 or more children. About 60% had only primary education or less and 76% were literate (could read and write very well). Less than a quarter (23%) were gainfully employed (work for which they were paid). About two-thirds started ANC in the second trimester and received more than four ANC visits. Most women received ANC from a nurse or midwife (88%) and solely from a health center or dispensary (55%). About 10% received ANC solely from a private facility and 34% received some ANC from a hospital. Eighty-eight percent went for their first ANC visit for a checkup (routine ANC), but 46% experienced some complication during the pregnancy and 31% felt the problem they had was severe. Table 2 and Table 3 shows the distribution of individual ANC quality measures for both service provision and experience of care.

**Table 1:** Sample distribution

	No.	*%*
Age		
15 to 19 years	177	17.2
20 to 29 years	599	58.1
30 to 48 years	255	24.7
Current marital status^a^		
Single	154	15
Partnered/Cohabiting	4	0.4
Married	811	78.7
Widowed	48	4.7
Divorced/Separated	13	1.3
Number of births^a^		
1	320	31.2
2	207	20.2
3	191	18.6
4 or more	309	30.1
Highest education		
No school/Primary	623	60.4
Post-primary/vocational/Secondary	292	28.3
College or above	116	11.3
Literacy: reading and write very well		
No	244	23.7
Yes	787	76.3
Employed with income		
No	792	76.8
Yes	239	23.2
Self or household member work in health facility		
No	967	93.8
Yes	64	6.2
Household wealth quintile^a^		
Poorest	247	24.2
Poorer	231	22.6
Middle	159	15.6
Richer	188	18.4
Richest	197	19.3
Household wealth quintile		
Poorest/Poorer	478	46.8
Middle	159	15.6
Richer/Richest	385	37.7
Current occupation		
Agricultural labor	170	16.5
Casual labor	63	6.1
Salaried worker	97	9.4
Self-employed in petty trade	189	18.3
Self-employed small-scale industry	29	2.8
Unemployed/homemaker	470	45.6
Other	13	1.3
Partner’s occupation^a^		
Agricultural labor	213	20.7
Casual labor	185	18
Salaried worker	157	15.3
Self-employed in petty trade	144	14
Self-employed small-scale industry	85	8.3
Unemployed/homemaker	25	2.4
Other	4	0.4
No Partner	215	20.9
Partner’s education^a^		
No school/Primary	397	39.3
Post-primary/vocational/Secondary	250	24.8
College or above	147	14.6
No Partner	215	21.3
Has health insurance^a^		
No	866	84.2
Yes	162	15.8
Tribe^a^		
Luo	696	67.6
Kuria	239	23.2
Other	95	9.2
Religious affiliation		
Catholic	271	26.3
Protestant/Pentecostal	233	22.6
Seventh Day Adventist	299	29
Other Christian	208	20.2
Muslim/other religion	20	1.9
Attitude towards domestic violence		
Tolerant	490	47.5
Intolerant	541	52.5
Participation in household decisions		
Low participation	531	51.5
High participation	500	48.5
Experienced domestic violence		
No	488	47.3
Yes	543	52.7
Had any pregnancy complications		
No	559	54.2
Yes	472	45.8
Had severe pregnancy complications		
No	709	68.8
Yes	322	31.2
Had complications in prior pregnancy		
No	894	86.7
Yes	137	13.3
Received ANC in prior pregnancy		
No	339	32.9
Yes	692	67.1
Prior facility delivery		
No	398	38.6
Yes	633	61.4
Highest ANC facility		
Gov’t Hospital	354	34.3
Gov’t HC/Dispensary	571	55.4
Mission/Private facility	106	10.3
Highest ANC Provider type		
Nurse/Midwife	905	87.8
Doctor/Clinical officer	115	11.2
Non-skilled attendant	11	1.1
Reason for first ANC^a^		
Because of a problem	112	10.9
Just for a checkup	909	88.3
Can’t Remember	9	0.9
Timing of first antenatal visit		
First trimester	300	29.1
Second trimester	634	61.5
Third Trimester	97	9.4
Number of antenatal visits^a^		
Less than 4	368	35.8
4 or 5	547	53.3
6 plus	112	10.9
Place of interview		
Health facility	421	40.8
In the community/a home	610	59.2
Postpartum length		
less than 1 week	81	7.9
1 week or more	950	92.1
N	1031	100

Notes: All totals equal 1031 except where noted by an ^a^; these have total of < 1031 due to missing data

**Table 2:** Distribution of quality of antenatal care variables

*Service provision*	No.	%
Height measured		
No	406	39.4
Yes	616	59.7
Don’t know or can’t remember	9	0.9
Weighed		
No, Never	22	2.1
Yes, A Few Times	93	9
Yes, Most Of The Time	86	8.3
Yes, All The Time	828	80.3
Don’t know or can’t remember	2	0.2
Blood pressure taken		
No, Never	99	9.6
Yes, A Few Times	207	20.1
Yes, Most Of The Time	122	11.8
Yes, All The Time	594	57.6
Don’t know or can’t remember	9	0.9
Did urine test		
No, Never	223	21.6
Yes, A Few Times	632	61.3
Yes, Most Of The Time	31	3
Yes, All The Time	139	13.5
Don’t know or can’t remember	6	0.6
Did a blood test		
No	35	3.4
Yes, once	789	76.5
Yes, more than once	207	20.1
Received a tetanus injection		
No	130	12.6
Yes	892	86.5
Don’t know or can’t remember	9	0.9
Iron supplementation		
No	98	9.5
Yes	915	88.7
Don’t know or can’t remember	18	1.7
Antihelminthes		
No	379	36.8
Yes	598	58
Don’t know or can’t remember	54	5.2
Antimalarials^a^		
No	162	15.7
Yes	849	82.4
Don’t know or can’t remember	19	1.8
Ultrasound^a^		
No	862	83.7
Yes	167	16.2
Don’t know or can’t remember	1	0.1
Total	1031	100

Notes: All totals equal 1031 except where noted by an ^a^; these have total of < 1031 due to missing data

**Table 3:** Distribution of quality of antenatal care variables

*Experience of care*	No.	%
Told the results after weighing^a^		
No, Never	103	10.2
Yes, A Few Times	82	8.2
Yes, Most Of The Time	104	10.3
Yes, All The Time	711	70.7
Don’t know or can’t remember	6	0.6
Told results after blood pressure measurements^a^		
No, Never	187	20.3
Yes, A Few Times	134	14.5
Yes, Most Of The Time	90	9.8
Yes, All The Time	493	53.4
Don’t know or can’t remember	19	2.1
Told results after urine test^a^		
No, Never	115	14.4
Yes, A Few Times	329	41.1
Yes, Most Of The Time	46	5.7
Yes, All The Time	303	37.8
Don’t know or can’t remember	8	1
Told results after blood test^a^		
No, Never	62	6.2
Yes, A Few Times	304	30.5
Yes, Most Of The Time	62	6.2
Yes, All The Time	554	55.6
Don’t know or can’t remember	14	1.4
Told about the signs of pregnancy complications^a^		
No	537	52.2
Yes	485	47.1
Don’t know or can’t remember	7	0.7
Told where to go in case of complications^a^		
No	445	43.2
Yes	582	56.5
Don’t know or can’t remember	3	0.3
Told what to expect during pregnancy and delivery		
No	566	54.9
Yes	458	44.4
Don’t know or can’t remember	7	0.7
Birth preparedness education^a^		
No	233	22.6
Yes	793	77
Don’t know or can’t remember	4	0.4
Nutrition education		
No	329	31.9
Yes	691	67
Don’t know or can’t remember	11	1.1
Breastfeeding education^a^		
No	365	35.4
Yes	655	63.6
Don’t know or can’t remember	10	1
Understood purpose of tests performed		
No, Never	170	16.5
Yes, A Few Times	178	17.3
Yes, Most Of The Time	234	22.7
Yes, All The Time	442	42.9
Don’t know or can’t remember	7	0.7
Understood purpose of medicines received		
No, Never	154	14.9
Yes, A Few Times	167	16.2
Yes, Most Of The Time	240	23.3
Yes, All The Time	462	44.8
Don’t know or can’t remember	8	0.8
Felt able to ask any questions^a^		
No, Never	178	17.3
Yes, A Few Times	216	21
Yes, Most Of The Time	195	19
Yes, All The Time	434	42.2
Don’t know or can’t remember	6	0.6
Asked if she had any questions^a^		
No, Never	306	29.7
Yes, A Few Times	206	20
Yes, Most Of The Time	153	14.9
Yes, All The Time	358	34.8
Don’t know or can’t remember	7	0.7
Felt treated with respect		
No, Never	15	1.5
Yes, A Few Times	82	8
Yes, Most Of The Time	230	22.3
Yes, All The Time	699	67.8
Don’t know or can’t remember	5	0.5
Treated in friendly manner^a^		
No, Never	25	2.4
Yes, A Few Times	109	10.6
Yes, Most Of The Time	247	24
Yes, All The Time	646	62.7
Don’t know or can’t remember	3	0.3
Could discuss issues in private		
No, Never	316	30.6
Yes, A Few Times	134	13
Yes, Most Of The Time	139	13.5
Yes, All The Time	438	42.5
Don’t know or can’t remember	4	0.4
Felt the health facility was clean		
No, Never	64	6.2
Yes, A Few Times	126	12.2
Yes, Most Of The Time	231	22.4
Yes, All The Time	599	58.1
Don’t know or can’t remember	11	1.1
Asked to give bribes^a^		
No, Never	912	88.5
Yes, A Few Times	64	6.2
Yes, Most Of The Time	29	2.8
Yes, All The Time	24	2.3
Don’t know or can’t remember	1	0.1
Felt treated differently because of any personal attribute		
No, Never	965	93.7
Yes, A Few Times	36	3.5
Yes, Most Of The Time	10	1
Yes, All The Time	16	1.6
Don’t know or can’t remember	3	0.3
Total	1031	100

Notes: All totals equal 1031 except where noted by an ^a^; these have total of <1031 due to missing data. The last two items on bribes and differential treatment were not included in the summative score because they loaded poorly on the dominant factor in the factor analysis

#### Service provision

About 60% had their height measured during their ANC and 80% reported their weight was measured at every visit. While guidelines recommend blood pressure and urine test at every ANC visit, only 58% reported their blood pressure was taken at every visit, and only 14% reported a urine test at every visit. Almost all (97%) received a blood test at least once, with 20% receiving a blood test more than once. In terms of preventative interventions, about 87% received a tetanus injection, 89% received iron supplementation, 58% received deworming medicine, and 82% were given antimalarial drugs, all of which are recommend per Kenya and WHO ANC guidelines. Only 16% of the women received an ultrasound during ANC. The average service provision score is 10.9 (SD = 2.4; range = 1 to 16) out of 16.

#### Experience of care

Women were not consistently informed of the results of their examinations or about pregnancy and delivery: about 71% reported they were always told their results after weighing, 53% always told blood pressure results, 38% always told urine test results, and 56% always told blood test results. Similarly, only 47% were told about the signs of pregnancy complications, 57% told where to go in case of complications, and 44% told what to expect during pregnancy and delivery. Over three-quarters (77%) reported receiving birth preparedness education, 67% nutrition education, and 64% breastfeeding education.

At least one out of three women reported sub-optimal understanding of ANC procedures: about 66% understood the purposes of tests performed most or all of the time and 68% understood the purposes of medicines received most or all of the time. Less than two-thirds of women (61%) felt they were able to ask questions most or all the time, and only 50% were consistently (most or all the time) asked if she had any questions.

Most women felt they were treated with respect most or all of the time (90%), and about 87% felt they were treated in a friendly manner most or all of the time. However, in terms of confidentiality, about 31% reported they could never discuss issues in private. Over half of the women (58%) felt the health facility was always clean. The majority of women were never asked to give a bribe (89%) and felt they were never treated differently because of any personal attribute (94%). These two measures were not included in the experience score index. The average experience score is 27.3 (SD = 8.2; range = 1 to 42) out of 42. The average score limited to the communication related items (excluding four questions on dignity and cleanliness) is 18.2 (SD = 6.8; range = 1 to 30) out of 30.

### Bivariate

Table 4 shows bivariate statistics for the association between the summative ANC quality measures and receipt of an ultrasound with various potential predictors. Significant differences exist in the ANC quality measures by sociodemographic factors as well as facility types. The following associations had *p*-values < 0.05. Not accounting for other factors, women who were older than 19 years and married women had, on average, higher experience of care scores than younger and unmarried women, respectively. Older women were also more likely to receive an ultrasound examination than younger women. Women with higher parity had lower service provision scores, including less likely to get an ultrasound. Compared to Luo women, Kuria women had higher experience scores and slightly higher service provision scores, but had lower odds of receiving an ultrasound.

**Table 4:** Bivariate regressions of antenatal care quality measures on various predictors, PQCC 2016/2017

	*Linear regression: Coefficient [95%CI]*						*Logistic regression: OR [95%CI]*		
	*Experience score*			*Service provision score*			*Received an ultrasound*		
Age									
15 to 19 years	0	[0	0]	0	[0	0]	1	[1	1]
20 to 29 years	1.98**	[0.55	3.41]	0.18	[-0.25	0.61]	2.40**	[1.34	4.32]
30 to 48 years	1.80*	[0.17	3.43]	-0.11	[-0.60	0.38]	2.84**	[1.51	5.31]
Marital status									
Single	0	[0	0]	0	[0	0]	1	[1	1]
Partnered/Cohabiting	-2.88	[-11.0	5.27]	-1.59	[-3.98	0.80]	1.38	[0.14	13.7]
Married	2.70***	[1.21	4.19]	-0.17	[-0.61	0.27]	0.77	[0.49	1.20]
Widowed	1.01	[-1.82	3.84]	-0.33	[-1.15	0.50]	0.59	[0.23	1.52]
Divorced/Separated	2.7	[-2.13	7.54]	0.18	[-1.30	1.66]	0.75	[0.16	3.57]
Number of births									
1	0	[0	0]	0	[0	0]	1	[1	1]
2	0.05	[-1.46	1.56]	-0.085	[-0.52	0.35]	1.4	[0.91	2.14]
3	0.16	[-1.38	1.69]	-0.52*	[-0.97	-0.069]	0.53*	[0.31	0.91]
4 or more	-0.65	[-2.01	0.70]	-1.08***	[-1.47	-0.68]	0.67	[0.43	1.04]
Tribe									
Luo	0	[0	0]	0	[0	0]	1	[1	1]
Kuria	3.96***	[2.71	5.20]	0.59**	[0.22	0.96]	0.28***	[0.16	0.49]
Other	1.2	[-0.61	3.01]	-0.2	[-0.75	0.35]	1.05	[0.62	1.80]
Religious affiliation									
Catholic	0	[0	0]	0	[0	0]	1	[1	1]
Protestant/Pentecostal	0.77	[-0.75	2.28]	-0.16	[-0.60	0.28]	0.58*	[0.36	0.93]
Seventh Day Adventist	0.55	[-0.87	1.98]	-0.11	[-0.52	0.30]	0.99	[0.66	1.48]
Other Christian	-0.46	[-1.99	1.06]	-0.58*	[-1.04	-0.13]	0.31***	[0.17	0.57]
Muslim/other religion	-2.48	[-6.42	1.45]	-1.21	[-2.42	0.0026]	0.2	[0.026	1.51]
Education									
No school/Primary	0	[0	0]	0	[0	0]	1	[1	1]
Post-primary/vocational/Secondary	2.15***	[0.96	3.34]	0.66***	[0.32	1.01]	2.03***	[1.35	3.04]
College or above	1.79*	[0.11	3.47]	1.17***	[0.69	1.66]	9.22***	[5.87	14.5]
Literate	2.28***	[1.05	3.51]	0.72***	[0.35	1.08]	2.57***	[1.57	4.19]
Employed	3.66***	[2.44	4.88]	0.86***	[0.50	1.22]	2.14***	[1.50	3.06]
Household wealth quintile									
Poorest/Poorer	0	[0	0]	0	[0	0]	1	[1	1]
Middle	0.73	[-0.80	2.26]	0.45	[-0.000034	0.90]	1.67	[0.93	3.01]
Richer/Richest	1.56**	[0.40	2.72]	0.78***	[0.44	1.12]	5.03***	[3.35	7.53]
Current occupation									
Agricultural labor	0	[0	0]	0	[0	0]	1	[1	1]
Casual labor	-0.71	[-3.18	1.77]	0.45	[-0.29	1.19]	1.86	[0.76	4.53]
Salaried worker	3.16**	[1.02	5.30]	0.79*	[0.17	1.42]	8.51***	[4.32	16.8]
Self-employed in petty trade	3.07***	[1.28	4.85]	0.53	[-0.00080	1.05]	2.10*	[1.07	4.12]
Self-employed small-scale industry	1.18	[-2.17	4.53]	0.28	[-0.72	1.28]	3.55*	[1.29	9.75]
Unemployed/homemaker	0.58	[-0.94	2.10]	0.19	[-0.26	0.64]	1.7	[0.93	3.13]
Other	1.31	[-3.92	6.54]	-0.017	[-1.72	1.69]	3.34	[0.82	13.6]
Partner’s education									
No school/Primary	0	[0	0]	0	[0	0]	1	[1	1]
Post-primary/vocational/Secondary	1.1	[-0.26	2.46]	0.3	[-0.093	0.70]	1.74*	[1.05	2.89]
College or above	2.22**	[0.64	3.81]	1.25***	[0.79	1.72]	7.59***	[4.67	12.3]
No Partner	-1.54*	[-2.96	-0.12]	0.44*	[0.025	0.85]	2.36***	[1.43	3.89]
Partner’s occupation									
Agricultural labor	0	[0	0]	0	[0	0]	1	[1	1]
Casual labor	-1.68	[-3.36	0.0057]	0.15	[-0.36	0.65]	1.32	[0.68	2.53]
Salaried worker	1.29	[-0.45	3.03]	0.91***	[0.38	1.43]	4.77***	[2.68	8.51]
Self-employed in petty trade	1.69	[-0.11	3.49]	0.51	[-0.029	1.04]	1.01	[0.48	2.12]
Self-employed small-scale industry	-0.74	[-2.90	1.41]	0.6	[-0.041	1.24]	2.19*	[1.05	4.54]
Unemployed/homemaker	-0.62	[-4.21	2.98]	0.17	[-0.91	1.25]	7.29***	[2.85	18.6]
Other	-4.75	[-12.8	3.31]	0.45	[-1.92	2.83]	1	[1	1]
No Partner	-2.23**	[-3.86	-0.60]	0.48*	[0.0028	0.97]	2.19**	[1.22	3.94]
Self or family work in health facility	2.95**	[0.74	5.17]	0.72*	[0.073	1.36]	1.97*	[1.10	3.51]
Has health insurance	3.08***	[1.66	4.50]	0.86***	[0.44	1.29]	3.45***	[2.36	5.05]
High participation in household decisions	1.30*	[0.25	2.35]	-0.011	[-0.32	0.30]	2.68***	[1.88	3.81]
Intolerant towards domestic violence	1.59**	[0.54	2.64]	0.86***	[0.55	1.17]	2.89***	[2.00	4.18]
Experienced domestic violence	-2.48***	[-3.53	-1.44]	-1.12***	[-1.42	-0.82]	0.55***	[0.39	0.77]
Had any pregnancy complications	0.84	[-0.22	1.90]	-0.40*	[-0.71	-0.092]	0.65*	[0.46	0.92]
Had severe pregnancy complications	1.45*	[0.31	2.59]	-0.27	[-0.61	0.061]	0.73	[0.50	1.06]
Had complications in prior pregnancy	-0.53	[-2.08	1.03]	-0.51*	[-0.98	-0.047]	0.93	[0.56	1.52]
Received ANC in prior pregnancy	-0.011	[-1.13	1.11]	-0.51**	[-0.84	-0.19]	0.9	[0.64	1.28]
Prior facility delivery	0.63	[-0.45	1.71]	-0.25	[-0.57	0.073]	1.04	[0.74	1.47]
Reason for first ANC									
Because of a problem	0	[0	0]	0	[0	0]	1	[1	1]
Just for a checkup	1.69*	[0.033	3.34]	0.077	[-0.43	0.59]	0.77	[0.47	1.27]
Can’t Remember	-4.45	[-10.0	1.13]	0.25	[-1.48	1.99]	0.51	[0.061	4.31]
Timing of first antenatal visit									
First trimester	0	[0	0]	0	[0	0]	1	[1	1]
Second trimester	-0.48	[-1.66	0.70]	-0.032	[-0.38	0.31]	0.87	[0.60	1.25]
Third Trimester	-3.31**	[-5.31	-1.31]	-1.36***	[-1.94	-0.78]	0.64	[0.33	1.26]
Number of antenatal visits									
Less than 4	0	[0	0]	0	[0	0]	1	[1	1]
4 or 5	1.50**	[0.37	2.64]	0.70***	[0.36	1.03]	1.57*	[1.07	2.29]
6 plus	2.34*	[0.53	4.15]	1.24***	[0.72	1.77]	1.75	[1.00	3.06]
Highest ANC facility									
Gov’t Hospital	0	[0	0]	0	[0	0]	1	[1	1]
Gov’t HC/Dispensary	0.68	[-0.46	1.81]	-0.52**	[-0.86	-0.19]	0.28***	[0.19	0.41]
Mission/Private facility	3.48***	[1.60	5.36]	0.5	[-0.045	1.05]	1.62*	[1.01	2.59]
Highest ANC Provider type									
Nurse/Midwife	0	[0	0]	0	[0	0]	1	[1	1]
Doctor/Clinical officer	1.37	[-0.27	3.02]	-0.16	[-0.66	0.34]	0.69	[0.38	1.24]
Non-skilled attendant	2.75	[-2.66	8.15]	-0.051	[-1.48	1.38]	1.11	[0.24	5.17]
In the community/a home	-2.19***	[-3.25	-1.13]	-0.58***	[-0.90	-0.26]	0.92	[0.65	1.28]
Postpartum length > =1 week	-2.97**	[-4.92	-1.03]	-0.88**	[-1.45	-0.31]	1.12	[0.59	2.12]

*Notes: 95% confidence intervals in brackets* * *p*<0.05 ** *p*<0.01 *** *p*<0.001

Women who were more empowered, from high SES households, had someone in their household working in a health facility, and had health insurance, had on average, higher experience of care scores compared to less empowered women, women from lower SES households, women who had no one in their household working in a health facility, and women who had no health insurance, respectively. The significance and direction of the associations between service provision and the empowerment and SES measures are similar. College educated and women from the wealthiest households have over five times higher odds of receiving an ultrasound than women with less than primary education and those from the poorest households.

Additionally, compared to women who had never experienced domestic violence, women who had experienced domestic violence had lower experience and service provision scores and had lower odds of getting an ultrasound. Also, women who had a severe pregnancy complication and first presented for ANC because of a problem had lower experience scores than those who had no severe pregnancy complication and first presented for ANC for checkup. Women who had any complication, however, had lower service provision scores, with lower odds of receiving an ultrasound.

Women who started ANC in the first trimester, received ANC four or more times, and solely from private facilities have higher experience scores than those who started ANC after the first trimester, received ANC less than four times, and from government facilities, respectively. Similarly, women who started ANC in the first trimester and who received ANC four or more times had higher service provision scores. Service provision scores, however, did not differ between government hospitals and private facilities, but were lower for health centers. Additionally, compared to women who received ANC services in hospitals, women who received ANC in health centers had lower odds of receiving an ultrasound, while those who received ANC solely in a private facility had higher odds of receiving an ultrasound.

### Multivariate

The multivariate models presented in Table 5 shows that, after controlling for other factors the following associations had *p*-values less than 0.05. On average, women in the 20 to 29 age group still have higher experience scores and those older than 30 years have higher service provision scores than those younger than 20 years. Both age groups are also over two times more likely to have done an ultrasound test than the younger women. Women with four or more children have lower service provision scores than the primiparous women. Net of other factors, Kuria women still had higher experience scores and slightly higher service provision scores, but had lower odds of receiving an ultrasound than Luo women.

**Table 5:** Multivariate regressions of antenatal care quality measures on various predictors, PQCC 2016/2017

	*Linear regression: Coefficient [95% CI]*						*Logistic regression: OR [95% CI]*		
	*Experience score*			*Service provision score*			*Received an ultrasound*		
Age									
15 to 19 years	0	[0	0]	0	[0	0]	1	[1	1]
20 to 29 years	1.72*	[0.088	3.35]	0.47	[-0.0069	0.96]	2.17*	[1.06	4.45]
30 to 48 years	1.78	[-0.35	3.91]	0.67*	[0.034	1.30]	2.92*	[1.21	7.04]
Currently married	2.94	[-4.57	10.5]	1.39	[-0.81	3.59]	0.55	[0.043	6.91]
Number of births									
1	0	[0	0]	0	[0	0]	1	[1	1]
2	-0.84	[-2.45	0.77]	-0.015	[-0.49	0.46]	1.26	[0.72	2.22]
3	-0.67	[-2.41	1.08]	-0.36	[-0.88	0.16]	0.52	[0.26	1.03]
4 or more	-0.88	[-2.74	0.99]	-0.71*	[-1.26	-0.16]	0.86	[0.43	1.72]
Tribe									
Luo	0	[0	0]	0	[0	0]	1	[1	1]
Kuria	4.97***	[3.70	6.25]	0.89***	[0.52	1.27]	0.37**	[0.20	0.69]
Other	1.57	[-0.16	3.31]	-0.068	[-0.59	0.45]	1.3	[0.70	2.41]
Literate	1.52*	[0.26	2.79]	0.22	[-0.16	0.60]	1.35	[0.76	2.38]
Employed	2.73***	[1.46	4.00]	0.56**	[0.19	0.93]	1.01	[0.64	1.59]
Participation in household decisions	1.24*	[0.14	2.34]	-0.16	[-0.49	0.17]	1.77*	[1.14	2.75]
Household wealth									
Poorest/poorer	0	[0	0]	0	[0	0]	1	[1	1]
Middle	0.99	[-0.49	2.47]	0.27	[-0.17	0.71]	1.03	[0.53	2.02]
Richer/richest	0.7	[-0.64	2.04]	0.13	[-0.27	0.53]	2.00**	[1.20	3.33]
Partner’s education									
No school/Primary	0	[0	0]	0	[0	0]	1	[1	1]
Post-primary/vocational/Secondary	0.16	[-1.21	1.52]	-0.062	[-0.47	0.35]	0.92	[0.52	1.64]
College or above	-0.46	[-2.27	1.36]	0.39	[-0.15	0.93]	2.40**	[1.29	4.46]
No Partner	1.42	[-6.20	9.03]	1.53	[-0.71	3.76]	0.94	[0.072	12.4]
Experienced domestic violence	-2.42***	[-3.51	-1.33]	-0.83***	[-1.15	-0.51]	0.91	[0.60	1.36]
Had severe complications	0.91	[-0.19	2.02]	-0.24	[-0.56	0.088]	1.19	[0.77	1.85]
Timing of first antenatal visit									
First trimester	0	[0	0]	0	[0	0]	1	[1	1]
Second trimester	-0.17	[-1.32	0.98]	0.16	[-0.19	0.50]	1.18	[0.76	1.81]
Third Trimester	-2.21*	[-4.28	-0.15]	-0.75*	[-1.36	-0.13]	0.82	[0.35	1.93]
Four plus antenatal visits	0.34	[-0.81	1.49]	0.38*	[0.033	0.72]	1.1	[0.70	1.74]
Highest ANC facility									
Gov’t Hospital	0	[0	0]	0	[0	0]	1	[1	1]
Gov’t HC/Dispensary	1.99***	[0.84	3.14]	-0.085	[-0.43	0.25]	0.33***	[0.21	0.52]
Mission/Private facility	3.28***	[1.48	5.09]	0.48	[-0.050	1.01]	1.31	[0.76	2.24]
Interviews in the community	-2.41***	[-3.44	-1.38]	-0.55***	[-0.86	-0.24]	1.08	[0.73	1.61]
Constant	20.9***	[13.1	28.6]	9.38***	[7.11	11.7]	0.08	[0.0056	1.16]
N	909			882			993		
R-squared	0.179			0.172					

95% confidence intervals in brackets * *p*<0.05 ** *p*<0.01 *** *p*<0.001

After controlling for other factors, women who are literate, employed, and participate in household decisions also still have higher experiences scores, but only employment is significant for higher service provision, and those with higher participation in household decision making are more likely to get an ultrasound. Household wealth and partner’s education are significantly associated with getting an ultrasound, with women from the wealthiest households and those with college educated husbands having about two times higher odds of receiving an ultrasound than women from the poorest households and whose husbands have less than primary education. Controlling for other factors, women who had experienced domestic violence still had lower experience and service provision scores than women who had never experienced domestic violence.

Women who started ANC in the third trimester had lower experience and service provision scores, and those who received ANC four or more times received slightly higher service provision scores. Timing and frequency of ANC is not significantly associated with odds of getting an ultrasound. Additionally, compared to women who received ANC in hospitals, women who received ANC in health centers had higher experience scores but lower odds of receiving an ultrasound, while those who received ANC solely in a private facility had higher experience scores, but no difference in service provision scores or the odds of getting an ultrasound. The effect of location of the interview persists after controlling for other factors.

## Discussion

This is one of the few studies to examine both service provision and experience dimensions of quality of ANC in a low resource setting, and to our knowledge, the first to do this at sub-national level in Kenya. We find that ANC quality is suboptimal in terms of providing recommended ANC services as well as ensuring women have a good experience. While many women receive basic ANC services such as blood pressure monitoring and urine test at least once during pregnancy, many are not receiving these consistently at every visit as recommended by the Kenya National guidelines [13]. The situation is even more dire for more advanced services such as ultrasounds, which less than one out of every five women in our sample received, with women who had complications (the group for whom it is recommended) less likely to receive it.

Although there is increased attention to mistreatment and poor person-centered care in facilities globally, most of this work has focused on intrapartum care. In this paper we also draw attention to poor PCANC, which can affect women’s adherence to treatment recommendations and deter them from returning to a facility to give birth [37–39]. The major gap in PCANC, which has been shown in other work for maternity care, is in the domain of communication [40]: women are not given sufficient information during ANC about their care, hence do not understand the purpose of examinations and medicines, but are not able to ask clarifying questions. Most women felt respected by their health care providers, which is encouraging. However, that 1 in 10 women did not feel respected means there is room for improvement, given the internalization and normalization of disrespect which usually results in low reporting when compared to observations [41–43]. We did not include survey questions on extreme forms of poor person-centered care such as verbal and physical abuse. But prior qualitative work in this setting suggests that verbal and physical abuse sometimes occurs during ANC [44].

As in many areas of health care, the most disadvantaged and disempowered women receive the lowest quality ANC relating to both service provision and experience of care. The potential reasons why more empowered and wealthier women are more likely to receive high quality ANC and person-centered care is described in detail elsewhere [20, 25, 40]. These reasons include being able to access care in facilities that offer higher quality care, being able to pay for higher quality care, and having the knowledge and ability to advocate for higher quality care. Studies in Kenya have shown that quality of care is poorer in low resource communities where poor women tend to live [22]. In this paper, we also find that the SES and empowerment differences are more marked for the experience of care dimensions than for the provision of ANC services. This is potentially because the services included in the service provision index are basic services that are offered free of charge to most clients, thus requiring less knowledge or ability to advocate for them.

The exception is in getting an ultrasound where SES measured by household wealth and partner’s education is a significant predictor. This finding is not surprising given the limited availability of ultrasounds in many government facilities in this setting. At the time of this survey, even the referral hospital had no functional ultrasound. Ultrasounds are also not covered by the National Health Insurance funds. This required that women who needed ultrasounds obtain it at private facilities where they had to pay before getting the services. Ultrasound costs in private facilities in Kenya vary widely (between 600 and 4000 Kenyan shillings (about 6 to 40 dollars) because of lack of regulation [45]. Furthermore, women with complications for whom ultrasound is recommended were less likely to receive an ultrasound than those with no complications. Therefore, those who need it the most may not be getting it because of cost due to systemic weaknesses. The higher odds of receiving an ultrasound among women who received some ANC in a hospital is likely because women receiving ANC in a hospital may have been referred there because of a complication, prompting providers there to request an ultrasound test. Ethnic differences between Luo and Kuria women in ANC quality might be due to biases against or in favor of certain ethnicities resulting in them receiving less services and being treated differently. We believe implicit bias plays an important role in quality of care differentials in this setting not just by ethnicity, but also based on SES and age, and thus account for some of those disparities too. Policies and interventions to improve quality of care therefore need consider how to address factors that contribute to these disparities.

The findings also suggest that certain high-risk women may not be getting key recommended services. For example, younger women (15 to 19 years) are less likely to get an ultrasound, in addition to being less likely have good PCANC. Given that this age group have high risk of complications, poor quality care may be playing a big role in their outcomes as complications may not be identified early or at all. In addition, perceptions of poor person-centered care may deter them from starting ANC early and attending frequently, further delaying identification of complications, and they may be less likely to deliver in a health facility where complications can be managed. Poor ANC quality in this group thus has detrimental consequences. Another high-risk group that was less likely to consistently receive the basic antenatal service was women with 4 or more children. This might be due to less attention to these women because of their prior childbirth experience, which could lead to adverse consequences for them if they receive less screening and preventative services. Other factors that account for differences in ANC quality are the timing and frequency of ANC. Both timing and frequency of ANC are important for the number of services one receives, but not for whether or not a woman gets an ultrasound. However, only timing is associated with experience of care, with women who received ANC in the third trimester reporting poorer experiences. This might be due to insufficient time for counselling and mistreatment from providers when women present for ANC late in the pregnancy, which we found in our qualitative work [44].

In addition, the types of facility where one receives care affects the quality of care they receive based on different dimensions. In general, there was no difference in service provision scores by facility. However, women who received ANC at least once from a government hospital had lower experience scores, but had higher odds of getting an ultrasound. On the other hand, those who received care in a health center, had higher experience scores, but had lower odds of getting an ultrasound. Women who received care in only a private facility also had higher experience scores, but had similar odds of getting an ultrasound as those who were seen in the government hospital. The finding of higher experience scores in health centers and private facilities is consistent with prior studies on women’s experiences for antenatal and delivery care and for family planning services [20, 40, 46, 47]. However, it raises the question of where the ‘best’ care for women might be during ANC [25]. Most women, particularly poor women, do not have the option of receiving care in private facilities. While they may receive more advanced essential services in the higher-level facilities, which have more staffing and clinical infrastructure, they also stand the risk of being mistreated in these facilities. Women should not have to choose between receipt of essential services and good person-centered care. Thus, there is a need for targeted PCANC interventions in higher-level facilities, as well as equipping the lower level facilities to be able to provide the essential antenatal services.

Various reasons, ranging from structural factors to provider attitudes, account for the suboptimal ANC quality. Providers will be unable to take weight and blood pressure measurements or to do blood and urine tests if they do not have working scales and blood pressure monitors or functional laboratories, reagents and supplies needed for these tests. Similarly, they will be unable to give medications they do not have in stock. Thus, availability of necessary equipment, supplies, and medicines are key to providing good quality ANC. These reasons are much more relevant for service provision than experience of care, although the frustration and stress of providing care without all the necessary tools could also manifest in providers’ interactions with women. Lack of provider knowledge of service provision guidelines and their knowledge and willingness to provide person-centered care is also a potential reason for poor quality. It is notable in the distribution of the individual measures shown in Table 2 that women were far more likely to be given various services than to be given information and listened to. One reason is that in ANC clinics where one provider may be trying to attend to several women, it is faster to do tests and dispense medication than spending time explaining to women and answering their questions. Thus, poor communication may be because of time constraints or workflow. The implication of this is that women might not be adhering to treatments and recommendations for further tests because they do not understand why these are necessary. Providers therefore need to be able to prioritize effective communication even in busy health facilities.

### Limitations

This study has potential limitations. Firstly, the measures of ANC quality are based on self-report. Recall bias is thus a potential limitation as women may not accurately remember whether or not they received a service. Second, although we assessed the appropriateness of combining the various items to generate the service provision and experience of care scores, the items are not from validated scales. Thus, there is a need for a more systematic process to developing validated scales for ANC service provision and experience of care. Additionally, in creating the summative scores, we coded ‘don’t know’/ ‘don’t remember’ responses as missing. But it is likely that women who said they can’t remember did not receive it, and if they don’t know, they likely weren’t told about it. Thus, we may have excluded women who received the poorest quality ANC, thus overestimating the actual levels of ANC quality. Social desirability bias is also potentially a limitation if women responded in a way that will please providers. This is likely a problem among women who were interviewed in a health facility and closer to the time of birth, as shown by the higher service provision and experience scores for women who were interviewed in a health facility and within a week of birth compared to women who were interviewed at home and after a week of birth. These are consistent with other findings on women’s experiences during childbirth [25, 26, 48]. Furthermore, we used proxy measures of empowerment, which may only partially address cognitive and psychological empowerment, and we are unable to account for structural factors that affect quality of care. Finally, the results are not generalizable to all of Kenya, as data was collected in a specific county using a multistage approach which included convenience samples within randomly selected health units.

Despite these limitations, this study makes valuable contributions to existing research on ANC quality in Kenya and other low-resource settings. It is among the few studies to examine both service provision and experience dimensions of quality of ANC in a low resource setting, thus extending the evidence base for calls to improve quality of ANC and person-centered care. Measuring the different dimensions of quality of care with several items enabled us to identify key areas that need to be addressed to improve quality of care. In addition, the use of composite measures enabled us to include multiple aspects of care provision and experience to assess quality of ANC on a continuum. Moreover, the inclusion of women who received care from different types of facilities enabled us to highlight issues that need to be considered at the different levels and types of facilities.

## Conclusions

This study adds to growing evidence on poor quality of maternal health care. We find that quality of ANC is suboptimal in both domains of service provision and experience of care, with disparities by demographic and socioeconomic factors as well as facility type. Much work is thus needed to improve both dimensions of quality of ANC at different levels of health facilities. In addition, disparities in quality of ANC based on demographic and social status need to be addressed in order to achieve the “no woman left behind” sustainable development goal. While it is still important to get women to health facilities, much more is needed to achieve the benefits of ANC, by ensuring that women consistently receive services required to prevent, identify, and manage complications. Furthermore, momentum for improving person-centered maternity care through the respectful maternity care movement should spread to ANC to ensure women are receiving person-centered care along the pregnancy childbirth continuum. As countries such as Kenya update their national guidelines for maternity care to align with new WHO standards, they must consider how to strengthen providers to provide person-centered care to all women in all types of facilities. Further research on the barriers and facilitators to providing high quality ANC will help guide further recommendations to improve quality of ANC and reduce disparities.

## Additional file

Additional file 1: Antenatal care questions from Perceived Quality of Care during Childbirth Study. (PDF 92 kb)

## Data Availability

The data analyzed for this manuscript are available from the corresponding author on reasonable request.

## Abbreviations

ANC: Antenatal care
PCANC: Person-centered antenatal care
WHO: World Health Organization
