# Supplementary material for: Quality of antenatal care and associated factors in a rural county in Kenya: an assessment of service provision and experience dimensions
Source: BMC Health Serv Res. 2019 Oct 7;19:684. doi: 10.1186/s12913-019-4476-4 (PMC6781384; doi:10.1186/s12913-019-4476-4)
Supplement: Supplementary file 1 — Antenatal care questions from Perceived Quality of Care during Childbirth Study. (PDF 92 kb) [file 12913_2019_4476_MOESM1_ESM.pdf]

Additional file 1: Quality of Antenatal Care Questions from Perceived Quality of Care during Childbirth Study, Kenya

**Now I am going to ask you about specific services that you received across all your antenatal visits. I know some of these are difficult to remember, so it is ok if you don't remember, but do try to tell me what you remember as it will be very useful in checking the quality of antenatal care provided in the facilities around here.**	
As part of your antenatal care during this pregnancy, were any of the following done:	
Was your height measured?	0, 0 No | 1, 1 Yes | 8, 8 Don't know or can't remember
Were you weighed?<br><br>(If yes) Were you weighed all the time, most of the time, or a few times?	0, 0 No, Never | 1, 1 Yes, A Few Times | 2, 2 Yes, Most Of The Time | 3, 3 Yes, All The Time | 8, 8 Don't Know Or Can't Remember
Were you told the results after you were weighed?<br><br>(If yes) Were you told the results all the time, most of the time, or a few times?	0, 0 No, Never | 1, 1 Yes, A Few Times | 2, 2 Yes, Most Of The Time | 3, 3 Yes, All The Time | 8, 8 Don't Know Or Can't Remember
Was your blood pressure taken?<br><br>(If yes) Was your blood pressure taken all the time, most of the time, or a few times?	0, 0 No, Never | 1, 1 Yes, A Few Times | 2, 2 Yes, Most Of The Time | 3, 3 Yes, All The Time | 8, 8 Don't Know Or Can't Remember
Were you told the results after your blood pressure was taken?<br><br>(If yes) Were you told the results all the time, most of the time, or a few times?	0, 0 No, Never | 1, 1 Yes, A Few Times | 2, 2 Yes, Most Of The Time | 3, 3 Yes, All The Time | 8, 8 Don't Know Or Can't Remember
Did you give a urine sample?<br><br>(If yes) Did you give a urine sample all the time, most of the time, or a few times?	0, 0 No, Never | 1, 1 Yes, A Few Times | 2, 2 Yes, Most Of The Time | 3, 3 Yes, All The Time | 8, 8 Don't Know Or Can't Remember
Were you told the results of the urine test?<br><br>(If yes) Were you told the results all the time, most of the time, or a few times?	0, 0 No, Never | 1, 1 Yes, A Few Times | 2, 2 Yes, Most Of The Time | 3, 3 Yes, All The Time | 8, 8 Don't Know Or Can't Remember

Did you give a blood sample ?<br><br>(If yes) Did you do a blood test once or more than once?	0, 0 No | 1, 1 Yes, Once | 2,2 Yes, More than once | 8, 8 Don't know or can't remember
Were you told the results of the blood test?<br><br>(If yes) Were you told the results all the time, most of the time, or a few times?	0, 0 No, Never | 1, 1 Yes, A Few Times | 2, 2 Yes, Most Of The Time | 3, 3 Yes, All The Time | 8, 8 Don't Know Or Can't Remember
During this pregnancy, were you given an injection in the arm to prevent the baby from getting tetanus, that is, convulsions after birth?	0, 0 No | 1, 1 Yes | 8, 8 Don't know or can't remember
During this pregnancy, were you given or did you buy any iron tablets or iron syrup?	0, 0 No | 1, 1 Yes | 8, 8 Don't know or can't remember
During this pregnancy, were you given or did you buy any drug for intestinal worms?	0, 0 No | 1, 1 Yes | 8, 8 Don't know or can't remember
During this pregnancy, were you given any drugs to keep you from getting malaria?	0, 0 No | 1, 1 Yes | 8, 8 Don't know or can't remember
During this pregnancy, did you do an Ultrasound?	0, 0 No | 1, 1 Yes | 8, 8 Don't know or can't remember
During any of your antenatal care visit(s):<br><br>Were you told about the signs of pregnancy complications?	0, 0 No | 1, 1 Yes | 8, 8 Don't know or can't remember
Were you told where to go if you had any complications?	0, 0 No | 1, 1 Yes | 8, 8 Don't know or can't remember
Were you ever told what to expect in the course of your pregnancy and delivery?	0, 0 No | 1, 1 Yes | 8, 8 Don't know or can't remember
During any of your antenatal care visit(s):<br><br>Did health provider ever talk to you about making prior arrangements for how to get to the health facility when you go into labor?	0, 0 No | 1, 1 Yes | 8, 8 Don't know or can't remember

Did a health provider ever talk to you about what to eat or how to eat well?	0, 0 No | 1, 1 Yes | 8, 8 Don't know or can't remember
Were you given any information or counseled about breast feeding?	0, 0 No | 1, 1 Yes | 8, 8 Don't know or can't remember
Thinking about all your antenatal care visits,<br>Did you feel the doctors, nurses or other staff treated you with respect?<br><br>(If yes) Will you say you were treated with respect all the time, most of the time, or only a few times?	0, 0 No, never | 1, 1 Yes, a few times | 2, 2 Yes, most of the time | 3, 3 Yes, all the time | 8, 8 Don't know or can't remember
Thinking about all your antenatal care visits,<br>Did you feel the doctors, nurses or other staff treated you in a friendly manner?<br><br>(If yes) Will you say you were treated in a friendly manner all the time, most of the time, or only a few times?	0, 0 No, never | 1, 1 Yes, a few times | 2, 2 Yes, most of the time | 3, 3 Yes, all the time | 8, 8 Don't know or can't remember
Thinking about all your antenatal care visits,<br>Do you feel you could discuss your problems with the doctors, nurses or other providers, without others not involved in your care overhearing your conversations?<br><br>(If yes) Will you say you had privacy all the time, most of the time, or only a few times?	0, 0 No, never | 1, 1 Yes, a few times | 2, 2 Yes, most of the time | 3, 3 Yes, all the time | 8, 8 Don't know or can't remember
Thinking about all your antenatal care visits, Did you feel you understood the purpose of any tests you were asked to do?<br><br>(If yes) Will you say this was all the time, most of the time, or only a few times?	0, 0 No, never | 1, 1 Yes, a few times | 2, 2 Yes, most of the time | 3, 3 Yes, all the time | 8, 8 Don't know or can't remember
Thinking about all your antenatal care visits, Did you feel you understood the purpose of any medicines you were given?<br><br>(If yes) Will you say this was all the time, most of the time, or only a few times?	0, 0 No, never | 1, 1 Yes, a few times | 2, 2 Yes, most of the time | 3, 3 Yes, all the time | 8, 8 Don't know or can't remember
Thinking about all your antenatal care visits, Did you feel you could ask the doctors, nurses or other staff at the facility any questions you had?<br><br>(If yes) Will you say this was all the time, most of the time, or only a few times?	0, 0 No, never | 1, 1 Yes, a few times | 2, 2 Yes, most of the time | 3, 3 Yes, all the time | 8, 8 Don't know or can't remember

Thinking about all your antenatal care visits, Did the doctors, nurses or other staff at the facility ask you if you had any any questions ?<br><br>(If yes) Will you say this was all the time, most of the time, or only a few times?	0, 0 No, never | 1, 1 Yes, a few times | 2, 2 Yes, most of the time | 3, 3 Yes, all the time | 8, 8 Don't know or can't remember
Thinking about all your antenatal care visits, Did any staff at the facility ask you for kitu kidogo?<br><br>(If yes) Will you say this was all the time, most of the time, or only a few times?	0, 0 No, never | 1, 1 Yes, a few times | 2, 2 Yes, most of the time | 3, 3 Yes, all the time | 8, 8 Don't know or can't remember
Thinking about all your antenatal care visits,<br>Would you say you were treated differently because of any personal attribute, like your age, marital status, number of children, your education, wealth, your connections with the facility, or something like that?<br><br>(If yes) Will you say this was all the time, most of the time, or only a few times?	0, 0 No, never | 1, 1 Yes, a few times | 2, 2 Yes, most of the time | 3, 3 Yes, all the time | 8, 8 Don't know or can't remember
Thinking about all your antenatal care visits, Did you feel the health facility environment, including the washrooms were clean?<br><br>(If yes) Will you say this was all the time, most of the time, or only a few times?	0, 0 No, never | 1, 1 Yes, a few times | 2, 2 Yes, most of the time | 3, 3 Yes, all the time | 8, 8 Don't know or can't remember
